# Be Careful

**Published:** 1880-08

**Authors:** 


### BE CAREFUL.

Be careful, young man, when you first commence business, to choose such an occupation, as will be the most conducive to health; for it will be more valuable to you than silver or gold. Be careful, young lady, how you trifle with life or death, as though they were of no value; the course you are pursuing will endanger them in a great many ways. You often, in damp weather, promenade the street, with thin soled shoes, regardless of the consequences; besides, you attend balls and parties, in winter, wearing that kind of apparel which is only appropriate for summer. Then, if you exercise, so as to be a little too warm, you forget to *be careful*, and often sit or stand in a draught of cold air, until perspiration is checked—then the " bad cold," often leading to a " fatal disease." Add to this, late hours from novel reading, and late rising, too, which prevents the morning breeze from blowing gently around you, to give you new life and animate your depressed spirits. It's true, you may go on in this way for months, and perhaps for a few years: but remember, the day of reckoning will come; then you would retract, but it may be too late; for by this time the " hollow cough" begins to startle you; but you indulge the hope that it's only a " cold," and form the resolution to be more careful for the future. But what is past can never be recalled. Your disease may now baffle the skill of the most eminent physicians. Despite the ceaseless watchings and prayers of a kind-hearted father and an affectionate mother, the king of diseases performs his task; your form becomes almost a shadow, and you sink into a premature grave. But how often disease might be prevented! if we would *be more careful*, and attend to the laws of health. While penning these lines, we are carried by retrospection to the days of our youth, when, if we had been guided more by judgment, we might now be enjoying better health. But like many others, we are now trying to *be careful*, and to profit by the advice we receive from our physician. Let us hope that the subject of health, at no distant day, will be looked at in its true light, and receive the attention it deserves and demands, and not be so often sold for a few transitory pleasures.

